# Anxiety and need for support of college students during the SARS-CoV-2 pandemic: An exploratory study

**DOI:** 10.1192/j.eurpsy.2021.791

**Published:** 2021-08-13

**Authors:** A. Torres, R. Melo, F. Príncipe, A. Ferreira, A. Quesado

**Affiliations:** 1 Cintesis - Pólo Ua, CINTESIS – Center for Health Technology and Services Research, Portugal (R&D Unit ref. UIDB/4255/2020), Aveiro, Portugal; 2 Nursing, Portuguese Red Cross Northern Health School, Oliveira de Azeméis, Portugal; 3 Center For Health Technology And Services Research (r&d Unit Ref. Uidb/4255/2020), CINTESIS, Porto, Portugal

**Keywords:** SARS-CoV-2, Anxiety, need for support, College students

## Abstract

**Introduction:**

During the pandemic state, college students are exposed to additional stressful factors, including but not limited to: fear of being infected; fear of infecting a significant person; deal with a new reality of economic uncertainty; challenges of distance education; new rules in face-to-face classes; restrictions on access to internships and higher demanding in internships.

**Objectives:**

This exploratory study aims to assess levels of anxiety and the perception of the need for support of students of a Portuguese Higher Education Institution (HEI).

**Methods:**

An exploratory study was developed, on the return of students to the presential classes after the academic lockdown. It was spread out an email for all students with a link for an online form, which includes sociodemographic questions, a screening question of the anxiety level, and the need for support level (rating scales 0-10).

**Results:**

It was obtained 36 answers from mostly female students (92%) with 17 to 21 years old (67%). Answers present an average anxiety level of 5.4 (Min=1; Max=9; SD=2.23), with 58% of answers with a score of anxiety level of 5 or higher. The need for support average was 3.7 (Min=1; Max=9; SD=2.23), with 33% of answers with a score of 5 or higher.

**Conclusions:**

It is necessary to continuously monitor the anxiety level and the need for support of college students during the SARS-Cov-2 pandemic. It is similarly relevant to have responses of HEI to promote mental health and to answer to the high levels of students’ anxiety and needs for support during the pandemic.

